# Supplementary material for: The interactome of intact mitochondria by cross-linking mass spectrometry provides evidence for coexisting respiratory supercomplexes
Source: Mol Cell Proteomics. 2017 Dec 8;17(2):216–32. doi: 10.1074/mcp.RA117.000470 (PMC5795388; doi:10.1074/mcp.RA117.000470)
Supplement: Supplemental Data [file supp_RA117.000470_133922_0_supp_23978_fzffwf.zip › spectra_annotation/mito_spectra_annotation/28-2-11-1-4-1.pdf]

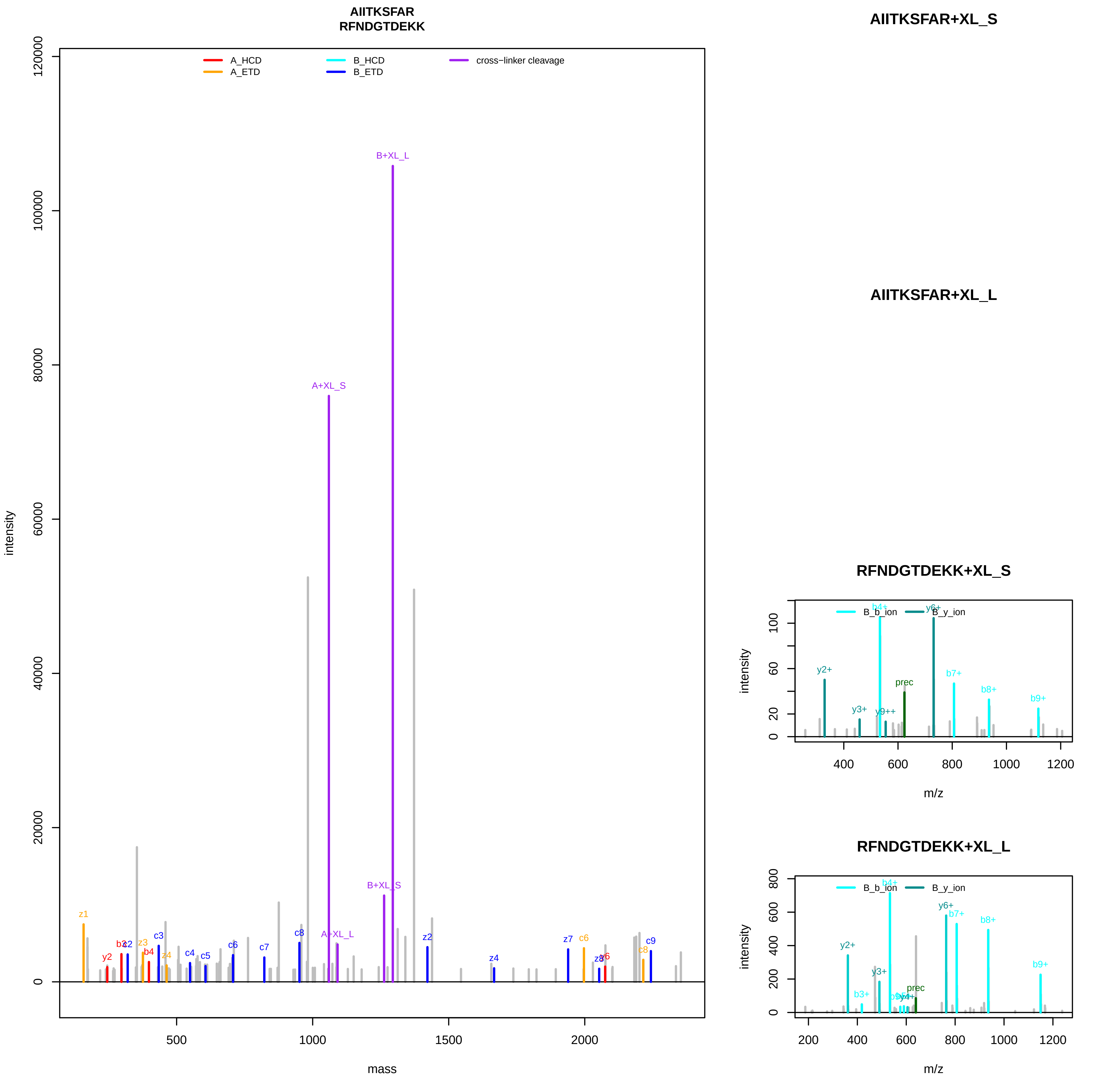
AIITKSFAR
RFNDGTDEKK
A_HCD
A_ETD
B_HCD
B_ETD
cross−linker cleavage
B+XL_L
A+XL_S
B+XL_S
A+XL_L
intensity
mass
0
20000
40000
60000
80000
100000
120000
500
1000
1500
2000
z1
y2
b3
c2
z3
b4
c3
z4
c4
c5
c6
c7
c8
z2
z4
z7
c6
z8
y6
c8
c9
AIITKSFAR+XL_S
AIITKSFAR+XL_L
RFNDGTDEKK+XL_S
B_b_ion
B_y_ion
b4+
y6+
y2+
prec
b7+
b8+
y3+
y9++
b9+
m/z
RFNDGTDEKK+XL_L
b4+
y6+
b7+
b8+
y2+
y3+
b3+
prec
y4+
b9+
m/z
